# ADAMTS2 gene dysregulation in T/myeloid mixed phenotype acute leukemia

**DOI:** 10.1186/1471-2407-14-963

**Published:** 2014-12-16

**Authors:** Giuseppina Tota, Nicoletta Coccaro, Antonella Zagaria, Luisa Anelli, Paola Casieri, Angelo Cellamare, Angela Minervini, Crescenzio Francesco Minervini, Claudia Brunetti, Luciana Impera, Paola Carluccio, Cosimo Cumbo, Giorgina Specchia, Francesco Albano

**Affiliations:** Department of Emergency and Organ Transplantation (D.E.T.O.) - Hematology Section, University of Bari, P.zza G. Cesare, 11, 70124 Bari, Italy

**Keywords:** Mixed phenotype acute leukemia, *ADAMTS2*, *TRD*, Complex chromosomal rearrangement, Promoter swapping, Gene dysregulation

## Abstract

**Background:**

Mixed phenotype acute leukemias (MPAL) include acute leukemias with blasts that express antigens of more than one lineage, with no clear evidence of myeloid or lymphoid lineage differentiation. T/myeloid (T/My) MPAL not otherwise specified (NOS) is a rare leukemia that expresses both T and myeloid antigens, accounting for less than 1% of all leukemias but 89% of T/My MPAL. From a molecular point of view, very limited data are available on T/My MPAL NOS.

**Case presentation:**

In this report we describe a T/My MPAL NOS case with a complex rearrangement involving chromosomes 5 and 14, resulting in overexpression of the ADAM metallopeptidase with thrombospondin type 1 motif, 2 (*ADAMTS2*) gene due to its juxtaposition to the T cell receptor delta (*TRD*) gene segment.

**Conclusion:**

Detailed molecular cytogenetic characterization of the complex rearrangement in the reported T/My MPAL case allowed us to observe *ADAMTS2* gene overexpression, identifying a molecular marker that may be useful for monitoring minimal residual disease. To our knowledge, this is the first evidence of gene dysregulation due to a chromosomal rearrangement in T/My MPAL NOS.

## Background

Mixed phenotype acute leukemias (MPAL) include acute leukemias with blasts that express antigens of more than one lineage, with no clear evidence of myeloid or lymphoid lineage differentiation [1]. Two provisional MPAL entities are defined, with regard to the association with the t(9;22)(q34;q11.2)/BCR-ABL1 and with the t(v;11q23)/MLL rearrangements. The term T/myeloid (T/My) and B/myeloid (B/My) MPAL not otherwise specified (NOS) refers to leukemia cases with both T or B and myeloid antigens, respectively, but without the above-mentioned genetic abnormalities [1]. T/My MPAL NOS is a rare leukemia accounting for less than 1% of all leukemias but 89% of T/My MPAL [2]; it can be seen in both children and adults and is generally considered to have poor prognosis [1, 2]. In a recent large series of patients affected by MPAL NOS, the most common abnormalities observed were complex karyotypes with a relatively frequent involvement of chromosomes 6q, 5 and 7 [2]. From a molecular point of view, data on T/My MPAL NOS are very scanty and limited. The most frequent cytogenetic abnormality in some T-cell disorders is the presence of chromosomal translocations with breakpoints in one of the T-cell receptor (TCR) loci [3]. As a result of this kind of rearrangement, the expression of the partner gene is dysregulated as the gene is placed under the transcriptional control of the TCR locus [4]. In this report we describe a T/My MPAL NOS case with a complex rearrangement involving chromosomes 5 and 14, resulting in overexpression of the ADAM metallopeptidase with thrombospondin type 1 motif, 2 (*ADAMTS2*) gene due to its juxtaposition to the T cell receptor delta (*TRD*) gene segment. To our knowledge, this is the first evidence of gene dysregulation due to a chromosomal rearrangement in T/My MPAL NOS.

## Case presentation

**Figure 1:**
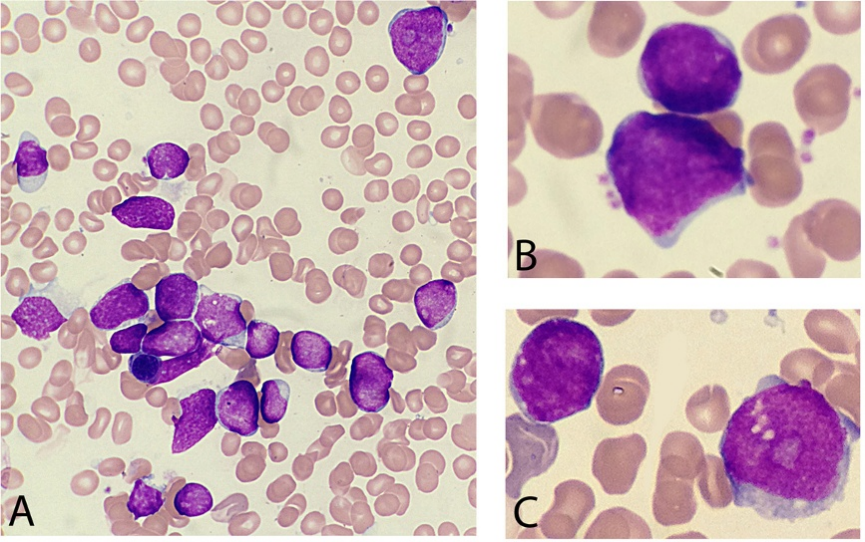
**May-Grunwald-Giemsa–stained bone marrow smear. (A)** A mixed-cell population of large and small blasts is observed. **(B-C)** Detail of different sized blast cells.

A young man aged 18 years was referred to our center for laterocervical lymphadenopathy and anemia. Peripheral blood smears analysis showed the presence of blast cells (19%). Bone marrow aspirate and biopsy confirmed diffuse blast infiltration (90%), characterized by a mixed-cell population of large and small blasts (Figure 1A-C). Immunophenotype analysis showed that blast cells were HLA-DR+, CD4+ CD5+, CD7+, cyCD3+, CD13+, CD33+, CD34+, MPO+, CD117+, CD56+. Conventional cytogenetic analysis of G-banded BM metaphase cells showed the following karyotype: 46,XY,?t(5;14)(q11;q11),?inv(6)(p11;p21)[9]/46,XY,idem,del(11q)[8]/46,XY[3]. Molecular analysis showed the occurrence of the FLT3 internal tandem mutation and the absence of the NPM1 mutation and BCR-ABL1 fusion gene. A diagnosis of T/My MPAL NOS was made according to the 2008 WHO criteria. The patient was treated with chemotherapy according to the Medical Research Council (UKALL XII)/ Eastern Cooperative Oncology Group (E2993) chemotherapy regimen [5]. After induction treatment the patient obtained complete hematological remission, which persisted during the consolidation and maintenance therapy. At these time points bone marrow immunophenotype analysis did not show the presence of blast cells. After sixteen months from the diagnosis, the patient is doing well and is still in hematologic, cytogenetic and molecular remission, defined by the *ADAMTS2* gene expression analysis.

**Figure 2:**
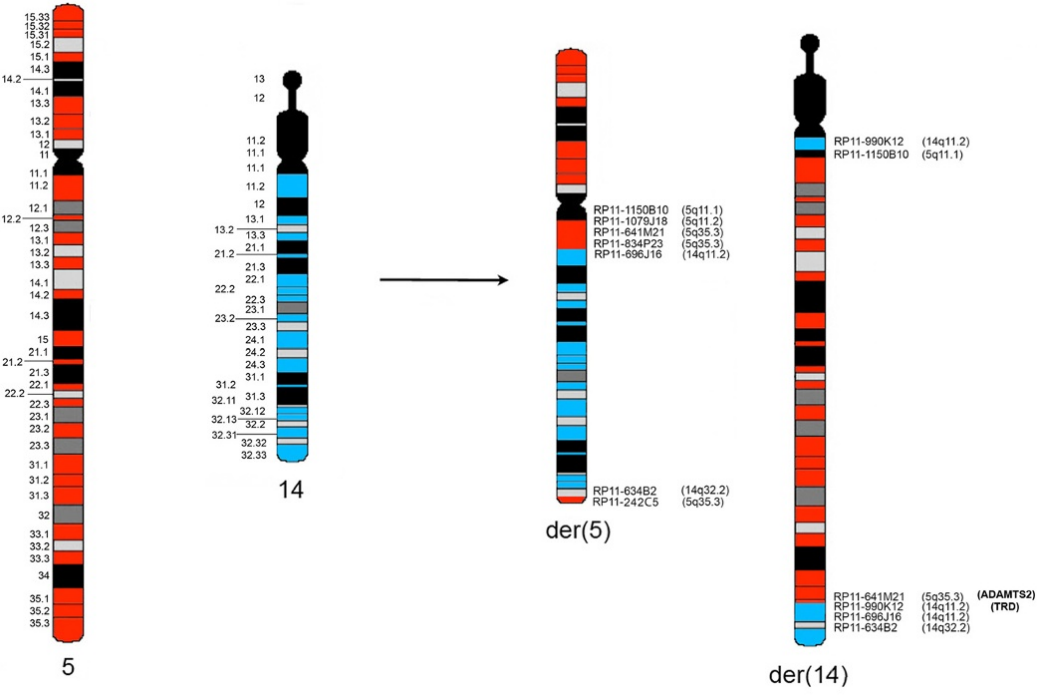
**Schematic representation of the complex chromosomes 5 and 14 chromosomal rearrangement.** The *ADAMTS2* gene was juxtaposed next to the *TRD* locus on der(14) in this case of T/My MPAL.

**Figure 3:**
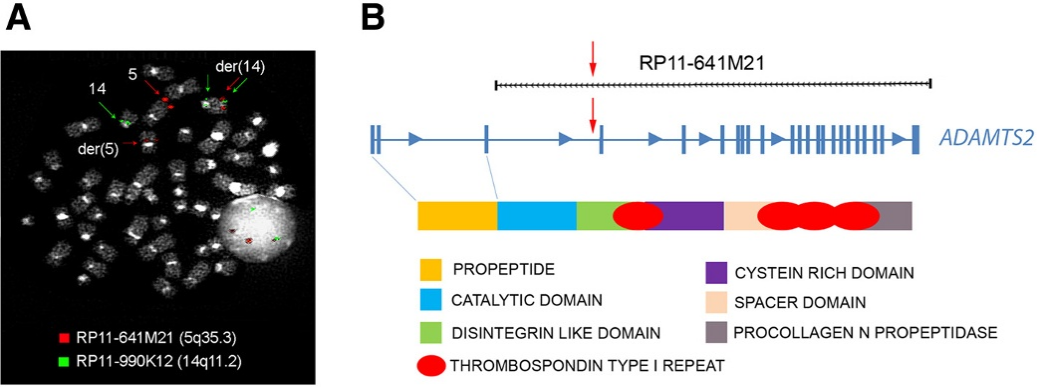
**FISH analysis and breakpoints identification. (A)** FISH cohybridization with BAC clones RP11-641 M21 (specific for the *ADAMTS2* gene) and RP11-990 K12 (specific for the *TRD* locus), identifying chromosomes 5 and 14 breakpoints, respectively. **(B)** The break in BAC RP11-641 M21 maps in the region included between exon 3 and 4 of the *ADAMTS2* gene (red arrow); exons 1–3 encode for the propeptide domain of the ADAMTS2 protein.

To confirm the conventional cytogenetic data regarding the t(5;14)(q11;q11) rearrangement, reiterative Fluorescence in situ hybridization (FISH) experiments with specific bacterial artificial chromosome (BAC) clones were carried out. FISH analyses were performed on BM samples, using BAC selected according to the University of California Santa Cruz database (UCSC http://genome.ucsc.edu/; Feb. 2009 release). Chromosome preparations were hybridized in situ with probes labeled by nick translation, as previously described [6]. FISH cohybridizations were done by chromosome walking, employing a total of 10 and 8 selected clones belonging to chromosomes 5 and 14, respectively (Table 1). According to the FISH pattern, a complex mechanism of double insertion with a concomitant duplication of the chromosome 5q pericentromeric region was hypothesized (Figure 2). In detail, chromosome 14 showed three different breakpoints: BAC clone RP11-990 K12 (chr14:22,860,409-23,039,541) was the insertion site of the chromosome 5 segment as it produced a signal on normal chromosome 14 and a splitting signal on der(14) (Figures 2 and 3A), whereas the region included between BAC clones RP11-696 J16 (chr14:23687161–23889944) and RP11-634B2 (chr14:99,566,527-99,778,328) was inserted in chromosome 5, as both clones showed a signal on normal chromosome 14 and a splitting signal on der(14) and der(5) (Figure 2). As for the chromosome 5 breakpoints, the region included between BAC clones RP11-641 M21 (chr5:178,525,062-178,697,496) (Figure 3A) and RP11-1150B10 (chr5:49406372–49441108) was inserted on der(14) as both clones hybridized on chromosomes 5, der(5) and der(14). Moreover, a duplicated region spanning from BAC RP11-1079 J18 (chr5:54403379–54555179) up to the centromere was identified on der(5). The insertion breakpoint of the chromosome 14 segment into chromosome 5 was mapped next to the chromosome 5 telomeric region between BAC clones RP11-834P23 (chr5:180585112–180782496) and RP11-242C5 (chr5:180720141–180862796) (Figure 2).

**Table 1:** **BAC clones employed in FISH experiments**

	BAC clones	Chromosomal band	Genomic position	FISH pattern
CHR 5	RP11-1149B6	5p11	chr5:46235286-46384443	5 + der(5)
	RP11-1150B10	5q11.1	chr5:49,406,372-49,441,108	5 + der(14) + der(5)
	RP11-1148I4	5q11.2	chr5:54,282,577-54,428,396	5 + der(14) + der(5)
	RP11-1079 J18	5q11.2	chr5:54403379-54555179	5 + der(14) + der(5)
	RP11-643H16	5q11.2	chr5:54612019-54764948	5 + der(14)
	RP11-699D5	5q32	chr5:148,691,900-148,899,365	5 + der(14)
	RP11-641 M21	5q35.3	chr5:178,525,062-178,697,496	5 + der(14) + der(5)
	RP11-994H18	5q35.3	chr5:180,532,073-180,720,900	5 + der(5)
	RP11-834P23	5q35.3	chr5:180,585,112-180,782,496	5 + der(5)
	RP11-242C5	5q35.3	chr5:180,720,141-180,862,796	5 + der(5)
CHR 14	RP11-614 K19	14q11.2	chr14:22,656,994-22,845,933	14 + der(14)
	RP11-990 K12	14q11.2	chr14:22,860,409-23,039,541	14 + der(14) splitting signal
	RP11-1083 M21	14q11.2	chr14:23068756-23274125	14 + der(14)
	RP11-909B16	14q11.2	chr14:23431729-23628645	14 + der(14)
	RP11-696 J16	14q11.2	chr14:23687161-23889944	14 + der(14) + der(5)
	RP11-828D3	14q11.2	chr14:23,897,700-24,068,595	14 + der(5)
	RP11-634B2	14q32.2	chr14:99,566,527-99,778,328	14 + der(14) + der(5)
	RP11-1145H5	14q32.33	chr14:106,995,083-107,135,809	14 + der(14)

After the achievement of complete hematologic remission, FISH analysis no longer revealed the presence of the chromosomal rearrangement. The lack of material precluded the possibility of investigating the other chromosomal abnormalities pointed out by the karyotypic analysis.

According to the UCSC database, the *ADAMTS2* and *TRD* genes were mapped in correspondence with the chromosomes 5 and 14 breakpoints, respectively. Therefore, as a consequence of the complex chromosomal rearrangement, the *ADAMTS2* gene was juxtaposed to the *TRD* locus on the der(14) chromosome. In particular, the FISH pattern data suggested that the breakpoint inside the *ADAMTS2* gene probably maps between exons 3 and 4 (Figure 3B). Bioinformatic analysis was performed by querying the Conserved Domain Database (http://www.ncbi.nlm.nih.gov/cdd ) for the annotation of functional domain proteins encoded by the coding sequence of the *ADAMTS2* gene. This analysis clarified that the exons 1–3, retained on der(5), code for the N-terminal propeptide domain of the metallopeptidase ADAMTS2; moreover, exons 4–22, transferred on der(14), encode for catalytic, disintegrin-like, cysteine-rich, spacer, 4 thrombospondin and procollagen N propeptidase domains (Figure 3B).

**Figure 4:**
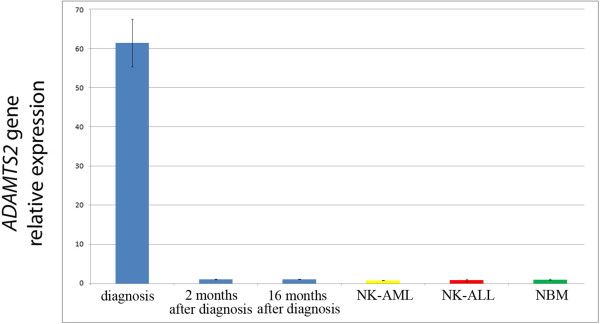
**qRT-PCR analysis.** Graphic representation of *ADAMTS2* gene relative expression evaluated in the T/My MPAL patient at onset, 2 and 16 months after the diagnosis (blue). The *ADAMTS2* gene expression in the pool of NK-AML and NK-ALL cases is indicated in yellow and red, respectively. Normal bone marrow (NBM) was employed as calibrator (green).

To verify the dysregulated expression of the *ADAMTS2* gene portion juxtaposed to the TRD locus on der(14), quantitative real-time PCR (qRT-PCR) analysis was carried out on the sample at diagnosis. Total RNA derived from BM cells was reverse transcribed into cDNA using the QuantiTect reverse transcription kit (Qiagen, Chatsworth, CA, USA). qRT-PCR experiments were carried out by using the LightCycler 480 SYBR Green I Master mix on the LightCycler 480II (Roche Diagnostics, Indianapolis, IN, USA). All samples were run in triplicate as technical replicates. The β-glucuronidase (*β-GUS*) gene was employed as endogenous control and a commercial pool of cDNA derived from healthy individuals BM cells (Clontech, US) was used as calibrator. Gene expression level was also compared to two pools of three normal karyotype (NK) AML and acute lymphoblastic leukemia (ALL) cases, respectively. Bone marrow samples from these patients were retrospectively obtained from the hospital archive. For *ADAMTS2* gene expression analysis, primers specific for exons 6 and 7 were selected according to the Primer3 software (http://frodo.wi.mit.edu) (forward primer: GCCACGATGAATACCACGAT, reverse primer: GGTGACAGGAGCATAGCCTTG). qRT-PCR experiments showed a higher expression level (61-fold change) compared to that of healthy bone marrow, NK-AML, and NK-ALL pools. qRT-PCR analysis was later performed at the time of complete hematologic and cytogenetic response and during the follow-up, revealing that by that time the *ADAMTS2* gene expression was not different from that observed in control sample (Figure 4).

## Discussion

The rarity of T/My MPAL NOS and the lack of application, in the cases described in literature, of the diagnostic criteria defined by the World Health Organization (WHO) classification, have made it difficult to establish whether this kind of leukemias has distinct biological features. In MPAL NOS no single karyotypic aberration was clearly recurrent, indicating that the leukemogenic process does not result from a specific genetic abnormality [2]. In this report we describe *ADAMTS2* gene overexpression following a complex rearrangement involving chromosomes 5 and 14 in a T/My MPAL NOS patient. This gene encodes a member of the ADAMTS proteins, a family of 19 secreted enzymes which has a role in extracellular matrix degradation and turn-over and has previously been involved in several biological processes such as cancer, coagulation, angiogenesis and cell migration [7]. Mutations in the *ADAMTS2* gene can induce the Ehlers-Danlos syndrome type VIIC, a recessive inherited connective-tissue disorder [8]. To date, there are no data about the link between ADAMTS2 function and leukemogenesis. ADAMTS2 has been reported to exert anti-angiogenic properties in vivo and in vitro through nucleolin, a nuclear protein also found to be associated with the cell membrane [9]. It is noteworthy that a recent study demonstrated significantly elevated nucleolin levels in acute myeloid leukemia (AML) patients, and that nucleolin overexpression was associated with DNA methyl transferase 1 upregulation and shorter survival [10]. Our report does not include data regarding nucleolin activity, but in the light of these data it can reasonably be argued that *ADAMTS2* overexpression may have a role in the leukemogenesis process. Although ADAMTS2 has been reported as tumour suppressor protein because of its anti-angiogenic properties [9], it is possible that a different function is carried out in hematopoietic tissue and that this function may be impaired, in our case, by the chromosomal rearrangement involving *ADAMTS2*. However, in our report no experimental validation has been performed to verify the leukemogenic potential of *ADAMTS2* gene as a consequence of the TCR locus juxtaposition and possibly constitutively metalloprotease activation.

It is interesting to note that the ADAMTS2 protein is physiologically synthesized as inactive zymogens, and its N-terminal propeptide is cleaved by furin, a proprotein convertase. This posttranslational modification is necessary for its activation. In this respect, in our case the *ADAMTS2* gene exons 1–3 encoding for the propeptide domain were retained on der(5) whereas the remaining *ADAMTS2* coding exons, that were found to be transferred adjacent to the *TCRD* locus on der(14), were overexpressed. Therefore, it is plausible that a rearrangement of the ADAMTS2 gene may be responsible for the production of an already active protein.

In T-cell ALL, up to 22 different oncogenes have been identified as *TCR* translocation partners; the *LMO2, TAL1,* and *TLX1* genes were most frequently involved in these rearrangements and the majority of all *TCR* translocations involved the *TRD* locus [3]. These aberrant recombinations result in juxtaposition of oncogenes in the vicinity of *TCR* cis-acting regulatory elements such as enhancers, which promote their expression [11]. In this respect our case showed three novel aspects: i) the first one is the fact that the *ADAMTS2* gene has never been described as a partner of rearrangement of the *TCR* locus; ii) the rearrangement between *ADAMTS2* and the *TRD* locus occurred through a mechanism of chromosomal insertion instead of translocation; iii) a dysregulated gene expression based on enhancer swapping [12, 13] has never been reported in T/My MPAL.

Data on the outcome of patients with MPAL are limited. Our patient was treated with the ALL-based regimen and achieved an optimal response. This observation confirmed previous findings arguing that ALL-based treatment seems more effective, with a higher response rate and better outcome, as compared with an AML or AML/ALL schedule [2, 14]. Apart from patient age, karyotypic and/or molecular associated aberrations may have a relevance, from the prognostic point of view, in T/My MPAL NOS.

## Conclusions

Detailed molecular cytogenetic characterization of the complex rearrangement in this T/My MPAL case allowed us to observe *ADAMTS2* gene overexpression, identifying a molecular marker that may be useful for monitoring minimal residual disease. In fact, the achievement of hematologic and cytogenetic response was associated with a normal *ADAMTS2* gene expression, comparable to that of healthy controls. In conclusion, considering the fact that T/My MPAL NOS is a disease whose biology and prognosis has not been completely defined because of its rarity, the possibility of carrying out molecular monitoring offers a great advantage, in order to be able to plan therapeutic treatments before it is possible to gain hematological or clinical evidence of leukemia relapse.

### Ethics statement

This study was performed in agreement with the Declaration of Helsinki, and approved by the local Ethical Committee (Comitato Etico Indipendente Locale, Azienda Ospedaliera “Ospedale Policlinico Consorziale” di Bari, Regione Puglia, prot. n. 324/C.E). Written informed consent was obtained from the patient for conducting molecular analysis and for publication of this Case report and any accompanying images. Written informed consent was also available from AML and ALL control patients for the use of tissues for molecular analyses and research purposes. A copy of written consents is available for review by the Editor of this journal.

## Abbreviations

ADAMTS2: ADAM metallopeptidase with thrombospondin type 1 motif, 2
AML: acute myeloid leukemia
ALL: acute lymphoblastic leukemia
BAC: bacterial artificial chromosome
B/My: B/myeloid
β-GUS: β-glucuronidase
BM: bone marrow
FISH: Fluorescence in situ hybridization
GTG-banded: G-banded with trypsin–Giemsa staining
MPAL: Mixed phenotype acute leukemia
NBM: normal bone marrow
NK: normal karyotype
qRT-PCR: quantitative real-time PCR
TCR: T-cell receptor
*TRD*: T cell receptor delta locus
T/My: T/myeloid
T/My MPAL NOS: T/myeloid Mixed phenotype acute leukemia not otherwise specified
UCSC: University of California Santa Cruz
WCP: whole chromosome painting
WHO: World Health Organization
